# Effects of Salinity Stress on Growth and Physiological Parameters and Related Gene Expression in Different Ecotypes of *Sesuvium portulacastrum* on Hainan Island

**DOI:** 10.3390/genes14071336

**Published:** 2023-06-25

**Authors:** Yong Wang, Wei Ma, Haijiang Fu, Liting Li, Xueyu Ruan, Xueyan Zhang

**Affiliations:** Ministry of Education Key Laboratory for Ecology of Tropical Islands, College of Life Sciences, Hainan Normal University, Haikou 571158, China; dhwang@nwsuaf.edu.cn (Y.W.); mawei19971109@163.com (W.M.); f_15764332567@163.com (H.F.); lilitingecnu@163.com (L.L.); ruanxeuyu18@163.com (X.R.)

**Keywords:** *Sesuvium portulacastrum*, salt stress, physiological parameters, gene expression

## Abstract

We conducted a study to examine the growth and physiological changes in 12 different ecotypes of *Sesuvium portulacastrum* collected from Hainan Island in China. These ecotypes were subjected to different concentrations (0, 200, 400, and 600 mmol/L) of sodium chloride (NaCl) salt stress for 14 days. We also analyzed the expression of metabolic genes related to stress response. Under low salt stress, indicators such as plant height in region K (0 mmol/L: 45% and highest at 200 mmol/L: 80%), internode length (0 mmol/L: 0.38, 200 mmol/L: 0.87, 400 mmol/L: 0.25, and 600 mmol/L: 1.35), as well as leaf area, relative water content, fresh weight, and dry weight exhibited an overall increasing trend with the increase in salt concentration. However, as the salt concentration increased, these indicators showed a decreasing trend. Proline and malondialdehyde contents increased with higher salt concentrations. When the NaCl concentration was 400 mmol/L, MDA content in the leaves was highest in the regions E (196.23%), F (94.28%), J (170.10%), and K (136.08%) as compared to the control group, respectively. Most materials demonstrated a significant decrease in chlorophyll a, chlorophyll b, and total chlorophyll content compared to the control group. Furthermore, the ratio of chlorophyll a to chlorophyll b (Rab) varied among different materials. Using principal component analysis, we identified three ecotypes (L from Xinglong Village, Danzhou City; B from Shuigoupo Village, Lingshui County; and J from Haidongfang Park, Dongfang City) that represented high, medium, and low salt tolerance levels, respectively, based on the above growth and physiological indexes. To further investigate the expression changes of related genes at the transcriptional level, we employed qRT-PCR. The results showed that the relative expression of *SpP5CS1*, *SpLOX1*, and *SpLOX1* genes increased with higher salt concentrations, which corresponded to the accumulation of proline and malondialdehyde content, respectively. However, the relative expression of *SpCHL1a* and *SpCHL1b* did not exhibit a consistent pattern. This study contributes to our understanding of the salt tolerance mechanism in the true halophyte *S. portulacastrum*, providing a solid theoretical foundation for further research in this field.

## 1. Introduction

*Sesuvium portulacastrum*, also known as Sea-purslane, belongs to the genus Sesuvium in the family Phyllanthaceae and is a succulent herbaceous saline plant that grows adnately in tropical and subtropical near-coastal sands and mudflats on both sides of river inlets, and is highly tolerant to abiotic stresses such as high salt, drought, and heavy metal ions [1]. *S. portulacastrum* is a perennial succulent herb with multi-branched, prostrate stems, 20–50 cm long, opposite, clasping leaves, flowers solitary in the leaf axils, flowers from April to July, and capsules with ovoid, shiny black seeds with raised tips [2]. It has strong salt tolerance and is a common Hainan coastal mangrove-associated true saline plant. It is valuable for both economic production and ecosystem remediation [3].

*S. portulacastrum* has a strong tolerance to adversity, with strong tolerance to both salt and heavy metal ions, and has the characteristics of compartmentalized enrichment of salt and heavy metal ions [4,5,6] and can grow and complete its life history in environments with varying salinity from freshwater to full seawater (about 0–600 mmol/L) [7] and its salt tolerance range is wide and to some extent it has salt-loving characteristics [8,9]. It was reported that the growth of *S. portulacastrum* was significantly inhibited with a 900 mmol/L NaCl salinity treatment [10] and, with an extremely high-salinity solution of 1000 mmol/L NaCl, it still survived for some time, but the growth was severely inhibited and some plants even died [11]. In addition, *S. portulacastrum* is tolerant to heavy metals such as lead, zinc, and cadmium [12]. Additionally, it has been found that low concentrations (5–10 μmol/L) of mercury can even promote plant growth and development [13]. This species also exhibits strong mercury tolerance and mercury enrichment properties relative to sweet-soil plants. Therefore, *S. portulacastrum* has a strong potential for environmental remediation of heavy metal pollution, coupled with fast reproduction, easy transplanting, easy survival, rapid growth, and high biomass, while showing strong adaptability to high temperatures, poor soils, and water bodies [14], which can be used for bioremediation of polluted habitats using ecological floating bed technology [13].

In the presence of high levels of salt stress, an excessive amount of energy is directed towards molecular oxygen, leading to the activation of oxygen poisoning through the overproduction of singlet oxygen, superoxide ion, hydrogen peroxide, and various other free oxygen radicals [15,16]. These free radicals have detrimental effects on proteins, lipids, nucleic acids, and other large molecules, causing damage [17,18]. High salt levels can lead to a series of changes in morphology, physiology, biochemistry, and molecular biology of cells and tissues in many plant species [19,20]. In past research work on *S. portulacastrum*, researchers obtained some physiological indicators related to stress resistance. The growth rate of *S. portulacastrum* and the effective nutrient content of culturewater may be positively correlated [21]. Han Bing et al. treated *S. portulacastrum* with Hoagland’s culture solution containing 0, 300, 600, and 900 mmol/L NaCl, and the Na^+^ content accumulated by *S. portulacastrum* leaves increased and K^+^ content decreased with the deepening of salt stress, and the Na^+^ content of *S. portulacastrum* could be effectively promoted in a certain NaCl concentration range and can effectively promote the growth and development of *S. portulacastrum* [22]. Previously, the free proline concentration and the activities of enzymes such as *SpP5CS*, *δ-OAT*, and *SOD* in *S. portulacastrum* under six different salt concentration stresses were reported, and it was concluded that the activities of enzymes such as δ-OAT, SpP5CS and SOD increased under salt stress, following the same trend as the accumulation of free proline. [23]. It has been documented that the isolated full-length cDNA sequence of *SpP5CS*, a key enzyme gene of the proline synthesis pathway from *S. portulacastrum*, was expressed significantly in *S. portulacastrum* leaves under high salt stress conditions, and the results indicated that the *SpP5CS* gene plays a key regulatory role [24]. In recent years, as the physiological changes and molecular mechanisms of resistance of *S. portulacastrum* under the stress of adversity have been intensively studied, its molecular mechanisms of salt tolerance have been gradually resolved [25], and more functions of *S. portulacastrum* salt tolerance genes have been verified by transgenic technology [26], which also provides valuable candidate genetic resources for subsequent molecular breeding of salt-tolerant crops. 

Chlorophyll degradation occurs when plants are subjected to biotic or abiotic stresses such as low temperature, bright light, pest and disease infestation, etc. and during the process of plant senescence, resulting in a series of physiological responses for self-protection, such as leaf fading, wilting, and early end of the life cycle [27]. It was found that chlorophyll degradation helps to recycle nitrogen sources from leaves that have completed their life history into new leaves or other nutrient organs required for reproductive growth through the chlorophyll degradation pathway [28]. Chlorophyllase (*CHL*) is the first enzyme for chlorophyll degradation and one of the earliest studied enzymes in plants and is widely found in higher plants, mosses, and algae. Numerous studies have demonstrated that the chlorophyllase 1 = of plants plays an important role in the chlorophyll degradation pathway [24,27,28,29,30,31,32,33].

In China, there is only one species of the genus Sesuvium, *S. portulacastrum* L. This species is mainly distributed on Hainan Island on coastal beaches, with scattered distributions on surrounding beaches and river inlets, but past studies used material from a regional source and did not systematically collect wild resources of *S. portulacastrum* from different ecotypes in the tropics. We obtained wild *S. portulacastrum* germplasm material from 16 representative localities through the systematic resource survey of *S. portulacastrum* in the coastal area of Hainan Island. The tropical ecotype of *S. portulacastrum* was found to be mainly distributed on sandy beaches at an average altitude of about 7.4 m above sea level, mainly located in the region of 18°15′ N to 20°05′ N and 108°39′ E to 110°59′ E on Hainan Island. Further identification confirmed that these 16 *S. portulacastrum* materials could be classified into 12 different ecotypes. Twelve distinct ecotypes of Sesuvium portulacastrum were exposed to salt stress using a monosaline (NaCl) method. This study aimed to examine the morphological and physiological modifications that occur in *S. portulacastrum* under salt stress. The expression variances of *P5CS*, *LOX*, and *CHL1* genes in *S. portulacastrum* were analyzed at various salt concentrations. The objective was to establish a theoretical framework for understanding the physiological and molecular mechanisms involved in salt tolerance in *S. portulacastrum*.

## 2. Materials and Methods

### 2.1. Plant Material and Growth Conditions

In this experiment, *S. portulacastrum* from 12 coastal areas of Hainan Island was used as the experimental material. There is a summary of the east longitude and north latitude of the sampling sites, together with physicochemical information for each site, in Table 1. For propagation, 6 cm long stem segments with two nodes and a pair of top leaves were cut from mother plants and cultured in 1/2 Hoagland medium [34]. After 3 weeks, when the adventitious roots start to grow from the nodes, the seedlings from 12 regions were treated with Hoagland nutrient solution with concentrations of 0 mmol/L (blank control), 200 mmol/L, 400 mmol/L, and 600 mmol/L NaCl for 14 days, respectively.

### 2.2. Morphological Measures

The water on the surface of the *S. portulacastrum* was roughly sucked up with absorbent paper and then the plant was spread on the table. The length from the root to the end of the terminal bud was measured with a ruler, that is, the whole length of the *S. portulacastrum*, and the height of the *S. portulacastrum* was recorded as shown in Figure S1. The internode length of the third and fourth nodes from the root of *S. portulacastrum* was measured with a ruler, which was recorded as the *S. portulacastrum* internode length. Plant height and internode length had at least three biological replicates. Each section of *S. portulacastrum* has two opposite leaves. The two opposite leaves in the third section from the root of *S. portulacastrum* were selected, the front and back sides of the two opposite leaves were measured by a leaf area scanner, and the average value of 8 measurements was taken as the final leaf area.

### 2.3. Determination of Relative Water Content (RWC)

The relative water content (RWC) of leaves for each treatment was calculated according to the formula of Weatherly [35].

RWC = [(fresh weight of leaves − the dry weight of leaves)/(turgid weight of leaves − dry weight of leaves)] ×100.

### 2.4. Determination of Fresh and Dry Weight

All *S. portulacastrum* material was taken from each treatment group in each area, and the plants were washed with water and wiped to dry their surfaces. Then, the fresh weight of the plants was weighed and recorded. The oven temperature was set to 105 °C, *S. portulacastrum* was transferred to the oven, then, 15 min later, the plants were taken out and the dry weight was measured.

### 2.5. Determination of Proline Content

Proline content was measured using the method described by [36], with modifications. First, 0.5 g of seedling shoot samples was ground in liquid nitrogen with a mortar and pestle, 10 mL of 3% sulfosalicylic acid was subsequently added, and the solution was then homogenized in a water bath at 99 °C. The supernatant was collected after centrifugation at 12,000× *g* for 10 min. Subsequently, 2 mL of glacial acetic acid and 3 mL of 2.5% ninhydrin reagent were added to 2 mL of supernatant. The mixture was incubated in boiling water for 60 min, and the reaction mixture was then extracted by adding 4 mL of methylbenzene. The absorbance of the extract liquor was determined at 520 nm, using a spectrophotometer.

### 2.6. Determination of Malondialdehyde (MDA)

MDA levels were determined by reaction with 2-thiobarbituric acid according to [37], with modifications. Briefly, fresh seedling shoot samples (0.3 g each) were homogenized and extracted in 10 mL of 0.25% TBA made in 10% trichloroacetic acid. The mixture was then heated in a water bath shaker at 95 °C for 30 min and rapidly cooled on ice. The absorbance of the supernatants was determined at 532 nm after centrifugation at 5000× *g* for 10 min. Correction of non-specific absorption was calculated by subtracting the absorbance at 600 nm. The concentration of MDA was expressed as μmol/g FW.

### 2.7. Determination of Chlorophyll Content

Chlorophyll content was determined following the method described by Arnon [38]. Fresh leaves, each containing 0.5 g of plant tissue, were extracted in the dark with 10 mL of 80% acetone. The assay mixture was centrifuged at 10,000× *g* for 5 min, and the supernatant was removed, mixed with 85% aqueous acetone solution to an appropriate concentration, and the absorbance was measured at 663 and 645 nm. Chlorophyll content was calculated according to the equations given by Arnon.

### 2.8. Total RNA Extraction and Reverse Transcription

An RNAprep Pure Plant kit (Tiangen, China, Beijing) was used to extract total RNA from 3 ecotypes after 14 days of salt stress (3 replicates were used from each treatment). Reverse transcription reactions were completed using 0.5 μg RNA and an iScript cDNA synthesis kit (Bio-Rad, Hercules, CA, USA). The ABI Power SYBR Green PCR Master Mix (Applied Biosystems, Waltham, MA, USA) was used with 5 ng of template. First-strand cDNA was synthesized from DNaseI-treated total RNA using 1 μL of iScript reverse transcriptase (TaKaRa, Gunma, Japan), 0.8 μg of RNA, and 4 μL of 5× Reaction Mix. Samples were incubated at 25 °C for 5 min, followed by 42 °C for 30 min, 85 °C for 5 min, and finally storage at 4 °C.

### 2.9. Acquisition of Gene-Coding Sequences

Based on the published genetic information of the proline synthase SpP5CS of *S. portulacastrum* [39], the SpP5CS protein sequence was derived from the results section of this paper. The protein sequences of *LOX1* and *LOX5* and the *CHL1* gene of Arabidopsis thaliana were downloaded from the Tair website. Candidate protein sequences (EVALUE < 1 × 10^−10^) were screened from *S. portulacastrum* transcriptome data using local BLAST.

The BioEdit software was used to compare the extracted protein sequences with the previously known sequences. Among them, the sequence of the common region was selected as the target sequence based on the extracted members of *SpP5CS* and named *SpP5CS1*. After sequence alignment, the two member sequences closest to *LOX1* and *LOX5* were selected as the target sequences and named *SpLOX1* and *SpLOX5*, respectively. The two sequences with the least significant difference from the *CHL1* sequences of Arabidopsis thaliana were selected as the target sequences and named *SpCHL1a* and *SpCHL1b*, respectively.

### 2.10. Quantitative Real-Time PCR (qRT-PCR)

The primer sequence information of the target sequence designed by Primer Premier 5 is shown in Table S1. Meanwhile, GAPDH was used as the reference gene in this study. The SYBR Green qRT-PCR reaction consisted of 5 μL of 2× SYBR Green PCR buffer, 0.5 μL of primers, and 5 ng of templates. The final volume was adjusted to 10 μL with double-distilled H_2_O. The PCR program was as follows: 50 °C for 2 min; 95 °C for 10 min; 40 cycles at 95 °C for 15 s and 60 °C for 1 min. Data were processed using the 2^−ΔΔCt^ method [40].

### 2.11. Data Analysis

Microsoft Excel 2010 was used for arranging the raw data and conducting calculations for the mean and standard deviation of both the control group and each treatment trait. To perform principal component analysis (PCA), SPSS 24.0 was employed.

### 2.12. A Comprehensive Evaluation of the Salt Resistance of Different Ecotypes

All indicators were used to determine change rates under salt stress. The salt resistance of different ecotypes of *S. portulacastrum* was comprehensively evaluated using principal component analysis.

## 3. Results

### 3.1. Effects of Salt Stress on Plant Height

**Figure 1:**
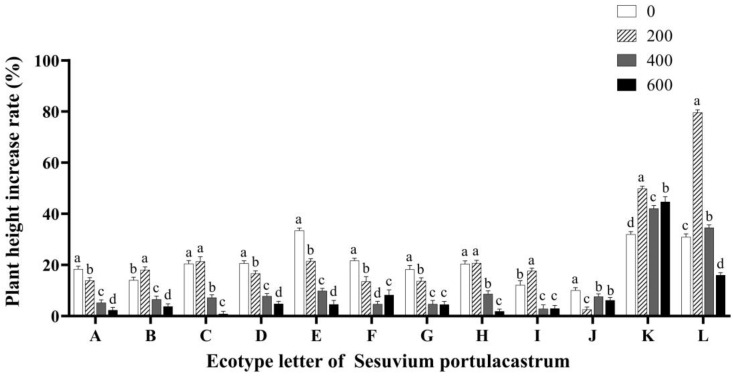
The effect of different concentrations of NaCl (0 mmol/L (blank control), 200 mmol/L, 400 mmol/L, and 600 mmol/L) on the growth rate of *Sesuvium portulacastrum* plant height from 12 coastal areas of Hainan Island (A–L). Note: Different lowercase letters only indicate significant differences in *Sesuvium portulacastrum* of the same ecotype under different salt concentration treatments (*p* < 0.05).

After 14 days of salt stress, different ecotypes of *S. portulacastrum* showed different growth statuses under different salt concentrations. The growth rate of *S. portulacastrum* in different regions showed significant differences. The growth rate of *S. portulacastrum* in regions A–J was low, while the growth rate of *S. portulacastrum* in regions K and L was good, and the growth rate was generally higher than that in other regions. For the same ecotypes of *S. portulacastrum*, the growth rate of plant height also showed great differences after different salt concentration treatments. After a 200 mmol/L salt treatment, the growth rate of *S. portulacastrum* height in B, I, K, and L was significantly higher than that in CK (*p* < 0.05). There was no significant difference in the growth rate of *S. portulacastrum* height between C and H compared with CK. Compared with the control group, the growth rate of plant height in other *S. portulacastrum* samples was significantly lower than that in CK. After the 400 mmol/L salt treatment, the growth rate of *S. portulacastrum* height in areas K and L was significantly lower than that in the 200 mmol/L salt treatment group (*p* < 0.05), and the growth rate of *S. portulacastrum* height in other places was significantly higher than that in the 200 mmol/L salt treatment group (*p* < 0.05). At the same time, under the stress of the 600 mmol/L salt concentration, some *S. portulacastrum* samples wilted and their growth was inhibited. However, the growth rate of *S. portulacastrum* height in area K was significantly higher than that in CK (*p* < 0.05), and higher than that in other regions with the same concentration treatment, and the growth rate of *S. portulacastrum* height was 44.71% (Figure 1).

### 3.2. Effects of Salt Stress on Internode Length

After 14 days of exposure to salt stress, the internode length of *S. portulacastrum* displayed significant variations across 12 different regions under varying salt concentrations. Notably, the control group exhibited more noticeable growth in regions F and I, measuring 0.74 cm and 0.61 cm, respectively. At a salt concentration of 200 mmol/L, regions C, D, H, K, and L demonstrated higher internode growth values, with the longest length observed in region K, reaching 0.87 cm. However, as the salt concentration increased to 400 mmol/L and 600 mmol/L, the growth rate of the internodes slowed down, resulting in relatively smaller elongation lengths. Furthermore, in some areas, the elongation process ceased altogether. Overall, the growth rate of internodes slowed down with the increase in salt concentration, which was consistent with the slow growth rate of *S. portulacastrum* with the increase in salt concentration. Among them, *S. portulacastrum* in region K were still elongating at the 600 mmol/L concentration, which was significantly different from the control group (*p* < 0.05), and the maximum length was 1.35 cm (Table 2).

### 3.3. Effects of Salt Stress on Leaf Area

**Figure 2:**
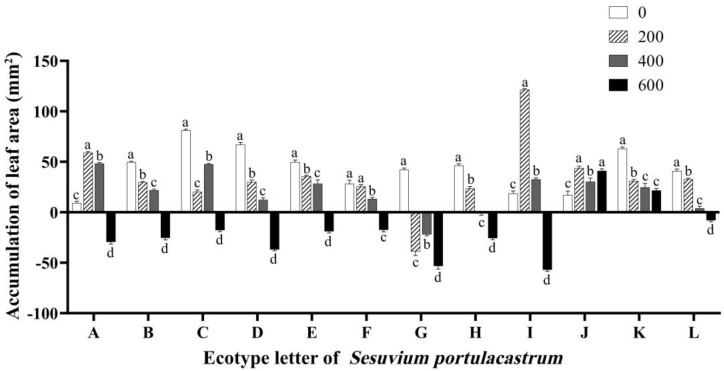
The effect of different concentrations of NaCl (0 mmol/L (blank control), 200 mmol/L, 400 mmol/L, and 600 mmol/L) on the growth of *Sesuvium portulacastrum* leaf area from 12 coastal areas of Hainan Island (A–L). Note: Different lowercase letters only indicate significant differences in *S. portulacastrum* of the same ecotype under different salt concentrations (*p* < 0.05), the same as below.

The experimental findings revealed that *S. portulacastrum* experienced an increase in leaf area in most areas under low salt concentration stress. However, when compared to the control group without stress, the stress impeded leaf growth. Specifically, in region I, the leaf area growth of *S. portulacastrum* was the highest at 121.44 mm^2^ when the salt concentration was 200 mmol/L. When the salt concentration reached 400 mmol/L, apart from regions G and H where the leaf area of *S. portulacastrum* showed negative growth values, the leaf area in other regions exhibited various degrees of growth. As the salt concentration further increased to 600 mmol/L, the leaf area of Sesuvium portulacastrum in all regions, except for regions J and K, displayed different degrees of reduction. At this time, the symptoms of shrinkage and etiolation of *S. portulacastrum* leaves appeared, and the growth value of the leaf area appeared negative. It is worth noting that *S. portulacastrum* in region G showed leaf wilting under 200 mmol/L to 600 mmol/L salt stress, which made leaf area smaller, and the value of the leaf area growth was significantly smaller than that of the control group (*p* < 0.05) (Figure 2). On the contrary, the leaf area of the J and K *S. portulacastrum* increased under all the salt concentration growth conditions (Figure 2).

### 3.4. Effects of Salt Stress on the Relative Water Content (RWC)

**Figure 3:**
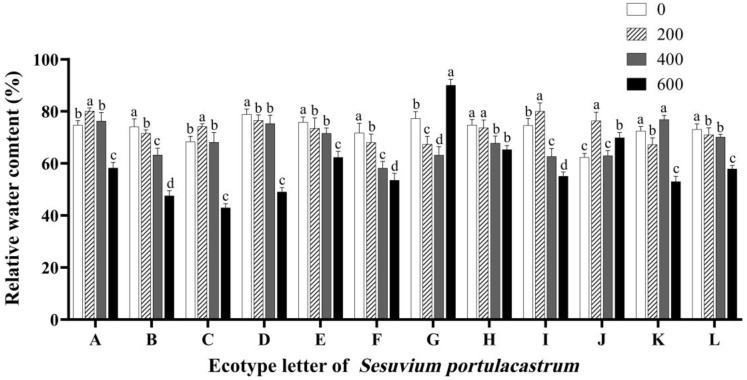
The effect of different concentrations of NaCl (0 mmol/L (blank control), 200 mmol/L, 400 mmol/L, and 600 mmol/L) on the relative water content of *S. portulacastrum* from 12 coastal areas of Hainan Island (A–L). Different lowercase letters in the same group indicate significant differences at *p* < 0.05.

RWC is a relatively sensitive index. The speed of decline in RWC under osmotic stress is closely related to plant stress resistance. Compared with absolute water content, it can better reflect the water status of leaves under osmotic stress (Figure 3). The RWC of *S. portulacastrum* in B, D, E, F, H, and L decreased with the increase in salt concentration and the RWC of the control group in area D was the highest, which was 78.86%. The RWC of *S. portulacastrum* in region J was higher than that of the blank control under all salt concentration treatments, and the maximum value was 76.43% when the salt concentration was 200 mmol/L. When the salt concentration was 0–400 mmol/L, the RWC of *S. portulacastrum* in region G decreased with the increase in salt concentration. When the salt concentration was 600 mmol/L, the RWC of *S. portulacastrum* in region G reached the highest value of 90%, which was significantly higher than that in other salt concentration treatment groups (*p* < 0.05). The RWC of *S. portulacastrum* in region K at 400 mmol/L salt concentration was significantly higher than that in other salt concentration treatment groups. The RWC of *S. portulacastrum* in other areas reached the maximum when the salt concentration was 200 mmol/L.

### 3.5. Effects of Salt Stress on the Fresh and Dry Weight

**Figure 4:**
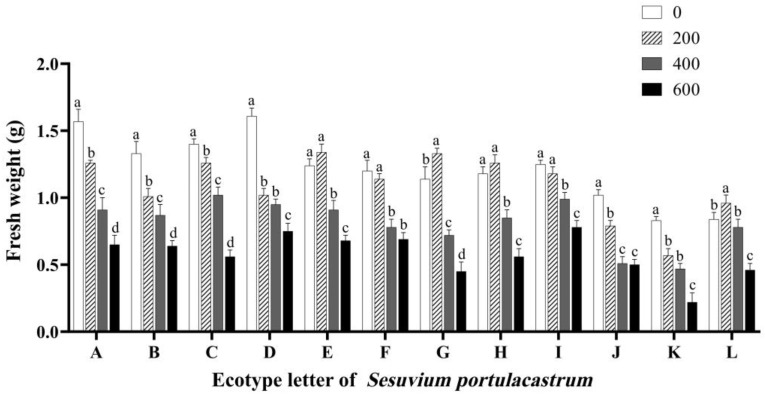
The effect of different concentrations of NaCl (0 mmol/L (blank control), 200 mmol/L, 400 mmol/L, and 600 mmol/L) on fresh weight of *S. portulacastrum* from 12 coastal areas of Hainan Island A–L. Different lowercase letters in the same group indicate significant differences at *p* < 0.05.

The most direct indicator of the effect of salt stress on plant growth is biomass. As shown in Figure 4, when the NaCl concentration was 200 mmol/L, the fresh weight of *S. portulacastrum* in some areas slightly increased compared with the control group. Among them, E increased by 8.12%, G increased by 16.03%, H increased by 6.02%, and L increased by 14.10% compared with the control. The fresh weight of *S. portulacastrum* in other areas decreased to different degrees. At the same time, the fresh weight of *S. portulacastrum* in most areas was greatly affected by the 400 mmol/L NaCl treatment. Except for that of L, which decreased to a lower degree, the fresh weight of *S. portulacastrum* in the other 11 areas decreased significantly compared with the control (*p* < 0.05), especially J, which decreased by 50% compared with the control. Compared with the control, K decreased by 43.12%, A decreased by 42.03%, and E decreased by 26.01%. When the concentration of NaCl reached 600 mmol/L, the *S. portulacastrum* in most areas was seriously injured, with yellowing, dehydration, and wilting, and the fresh weight was significantly lower than that of the control group. The overall fresh weight of *S. portulacastrum* decreased gradually with the increase in salt concentration.

**Figure 5:**
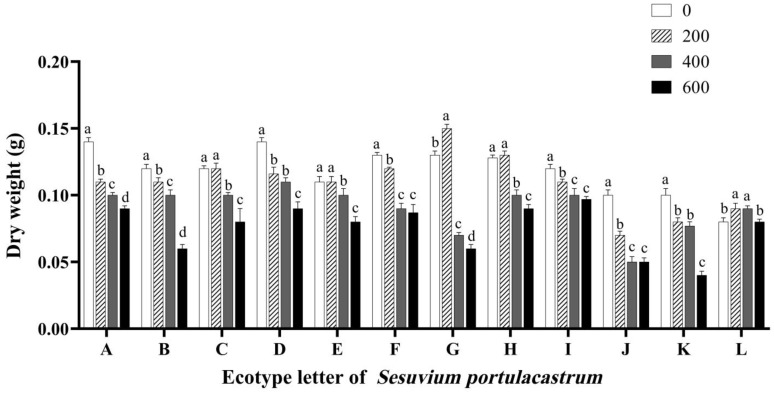
The effect of different concentrations of NaCl (0 mmol/L (blank control), 200 mmol/L, 400 mmol/L, and 600 mmol/L) on the dry weight of *S. portulacastrum* from 12 coastal areas of Hainan Island (A–L). Different lowercase letters in the same group indicate significant differences at *p* < 0.05.

As shown in Figure 5, the change in dry weight of *S. portulacastrum* was similar to the above trend of fresh weight. Under the 200 mmol/L NaCl treatment, the dry weight of *S. portulacastrum* in some areas was slightly higher than that of the control group treated with 0 mmol/L NaCl. Compared with the control group, E increased by 3.13%, G increased by 15.01%, H increased by 5.02%, L increased by 13.04%. The dry weight of *S. portulacastrum* in other areas decreased to different degrees. At the same time, under the 400 mmol/L NaCl treatment, the dry weight of *S. portulacastrum* in the other 11 sites was significantly decreased compared with the control, except the dry weight of L that was increased by 12.5% compared with the control (*p* < 0.05). G decreased by 46.14%, J decreased by 31.08%, and F decreased by 28.14% compared with the control. When the NaCl concentration reached 600 mmol/L, the dry weight of *S. portulacastrum* further decreased to different degrees in almost all places. The overall dry weight of *S. portulacastrum* decreased gradually with the increase in salt concentration.

The above results indicate that some *S. portulacastrum* grow faster in environments with a low concentration of NaCl. When the concentration of NaCl exceeds a certain range, the growth rate of *S. portulacastrum* will slow down, and the *S. portulacastrum* may become dehydrated and withered with the further increase in NaCl concentration. At the same time, we found that J was relatively more inhibited by NaCl in the *S. portulacastrum* materials from 12 regions, suggesting that this material was sensitive to salt stress. The dry weight of L was not significantly affected by NaCl and remained at a relatively stable level. Combined with the fact that its fresh weight increased at 200 mmol/L NaCl and showed the smallest decline in 400 mmol/L NaCl, it was speculated that *S. portulacastrum* samples in region L were not very sensitive to salt stress.

### 3.6. Effects of Salt Stress on Proline Content

**Figure 6:**
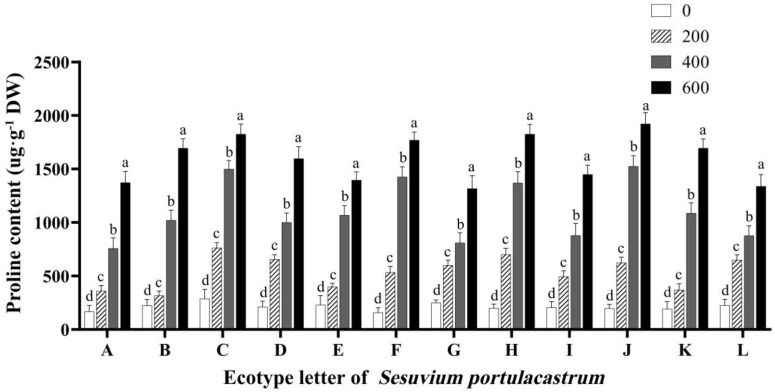
The effect of different concentrations of NaCl (0 mmol/L (blank control), 200 mmol/L, 400 mmol/L, and 600 mmol/L) on proline content in *S. portulacastrum* from 12 coastal areas of Hainan Island (A–L). Different lowercase letters in the same group indicate significant differences at *p* < 0.05.

As shown in Figure 6, when NaCl concentration was low, the proline content in *S. portulacastrum* leaves was low, and when NaCl concentration gradually increased, the proline content also showed a gradual increase. When NaCl concentration was 200 mmol/L, the proline content of *S. portulacastrum* leaves in A–L was significantly increased compared with the control group (*p* < 0.05). In addition to B and E, the proline content of dentate leaves of *S. portulacastrum* in other areas was increased by more than 2 times compared with the control. When NaCl concentration was 400 mmol/L, compared with the control group and 200 mmol/L treatment group, the content of proline in *S. portulacastrum* leaves was significantly increased (*p* < 0.05), and the increased amplitude was greatly increased. In particular, the proline content of F and J reached 7 times higher than that of the control group. After that, the concentration of NaCl was further increased. Under the treatment of 600 mmol/L NaCl, the proline content in the leaves of *S. portulacastrum* in some areas was nearly 10 times that of the control group, as shown in J.

### 3.7. Effects of Salt Stress on Malondialdehyde (MDA) Content

**Figure 7:**
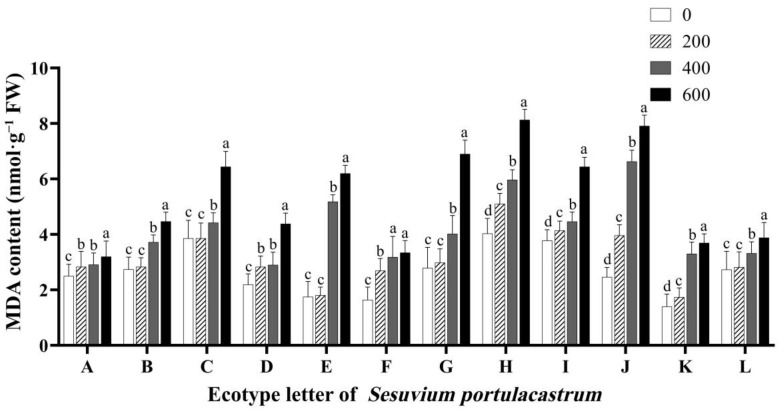
The effect of different concentrations of NaCl (0 mmol/L (blank control), 200 mmol/L, 400 mmol/L, and 600 mmol/L) on malondialdehyde in *S. portulacastrum* from 12 coastal areas of Hainan Island (A–L). Different lowercase letters in the same group indicate significant differences at *p* < 0.05.

MDA is the product of lipid metabolism, and its content can directly reflect the degree of stress-induced membrane damage. After 14 days of salt stress, the measurement results showed that MDA content in *S. portulacastrum* leaves in 12 regions showed an increasing trend with the increase in NaCl concentration (Figure 7). When the treatment condition was 200 mmol/L NaCl concentration, compared with the control group, the MDA content of *S. portulacastrum* leaves in each region was 13.02% (A), 3.20% (B), 0.02% (C), 29.18% (D), 3.15% (E), 64.75% (F), 7.52% (G), 27.26% (H), 10.25% (I), 61.34% (J), 24.07% (K), and 3.16% (L) higher. When the NaCl concentration was 400 mmol/L, MDA content in *S. portulacastrum* leaves in 12 regions was 16.75% (A), 36.28% (B), 15.32% (C), 32.18% (D), 196.23% (E), 94.28% (F), 44.32% (G), 48.24% (H), 18.15% (I), 170.10% (J), 136.08% (K), and 22.62% (L) higher than that in the control group. When the concentration of NaCl reached 600 mmol/L, MDA content in *S. portulacastrum* leaves in all regions was further increased, especially in E, G, H, and J, which had contents 254.29%, 147.31%, 101.74%m and 221.54% higher than those in the control group, respectively.

These results indicate that a high concentration of salt may lead to peroxidation of polyunsaturated fatty acids in the cell membrane of *S. portulacastrum* mesophyll, which may cause cell damage, damage the cell membrane structure, and increase membrane permeability. In conclusion, the accumulation of MDA in *S. portulacastrum* leaves under salt stress reflects the degree of injury. According to the above results, *S. portulacastrum* from the 12 regions can be divided into A, B, I, and L ecotypes with high salt tolerance, C, D, F, and K ecotypes with moderate salt tolerance, and E, G, H, and J ecotypes with low salt tolerance.

### 3.8. Effects of Salt Stress on Chlorophyll a Content

**Figure 8:**
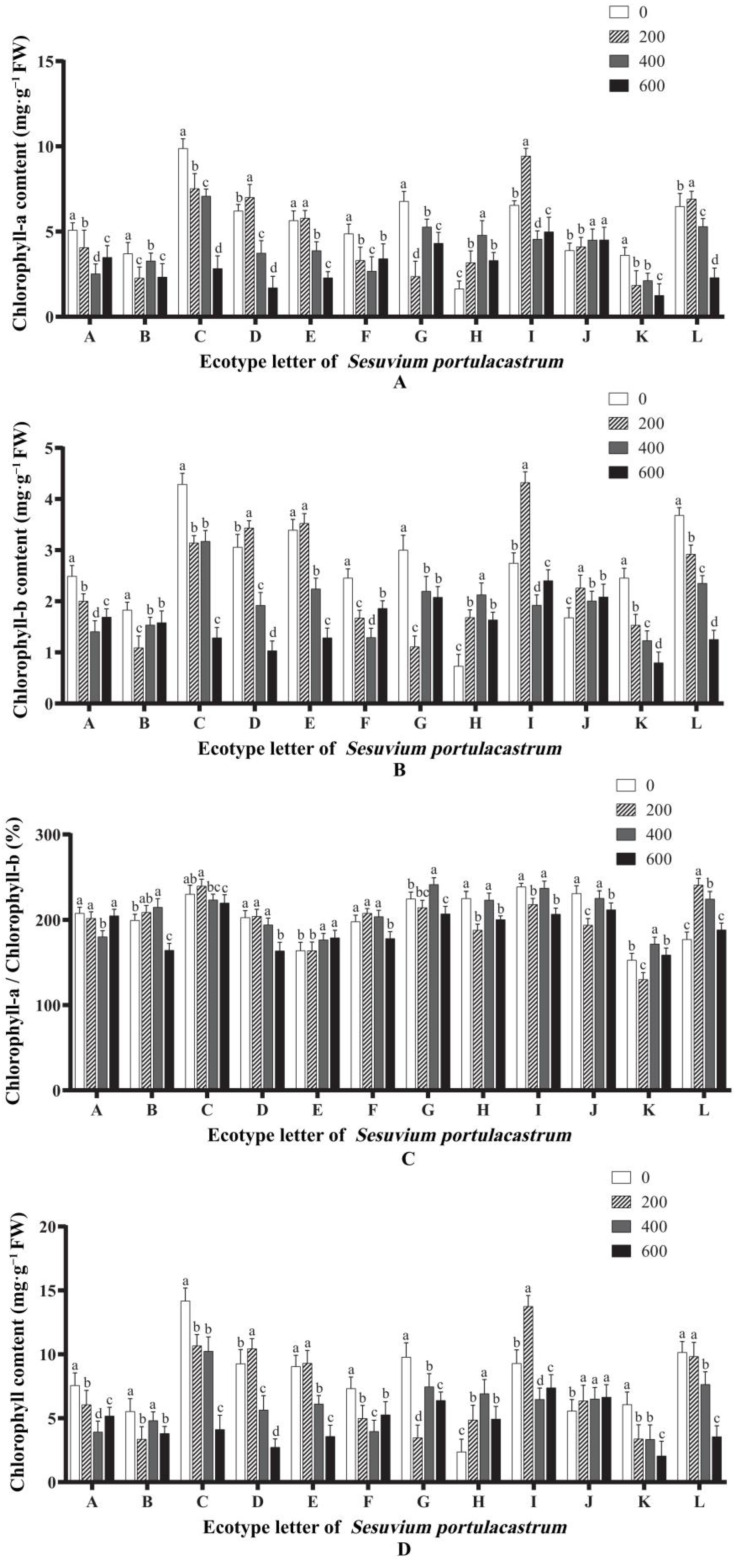
The effect of different concentrations of NaCl (0 mmol/L (blank control), 200 mmol/L, 400 mmol/L, and 600 mmol/L) on chlorophyll a content (**A**), chlorophyll b content (**B**), ratio of chlorophyll a to chlorophyll b (**C**), and total chlorophyll content (**D**) of *S. portulacastrum* from 12 coastal areas of Hainan Island (A–L). Different lowercase letters in the same group indicate significant differences at *p* < 0.05.

Figure 8A shows the changes in *Chla* content in 12 different ecotypes of *S. portulacastrum* leaves. When the concentration of NaCl was 200 mmol/L, the *Chla* content in *S. portulacastrum* leaves at E, J, and L had no significant change compared with that in the control group. D, H, and I had values that were significantly higher than that in the control group (*p* < 0.05), while the content of Chla in other places was significantly lower than that of the control group (*p* < 0.05). When NaCl concentration was 400 mmol/L, there was no significant difference in the content of *Chla* in the *S. portulacastrum* leaves of area B compared with the control group, the content of *Chla* in the *S. portulacastrum* leaves of areas H and J was significantly higher than that of the control group, and the content of *Chla* in other areas was significantly lower than that of the control group (*p* < 0.05). When salt concentration continued to rise to 600 mmol/L, the *Chla* content in *S. portulacastrum* leaves in only region J was significantly higher than that in control group, and the other regions had values that were significantly lower than that in the control group (*p* < 0.05). In general, the *Chla* content in *S. portulacastrum* leaves increased under low concentrations of salt stress in some areas. When the salt concentration continued to increase, the *Chla* content in *S. portulacastrum* leaves showed a decreasing trend, generally becoming lower than that in the control group (*p* < 0.05).

### 3.9. Effects of Salt Stress on Chlorophyll b Content

Valentina et al. demonstrated that the decrease in *Chlb* content under salt stress can reduce the capture of light energy, the production of reactive oxygen species, and the degradation rate of proteins, making plants more salt resistant. After 14 days of salt stress, the changes in *Chlb* content in *S. portulacastrum* of different ecotypes under different salt concentrations were similar to those of *Chla*. The changes in *Chlb* content in *S. portulacastrum* leaves of different regions showed significant differences, and the *Chlb* content in *S. portulacastrum* leaves of most regions under different salt concentrations was generally lower than that of the control group (Figure 8B). The *Chlb* content of *S. portulacastrum* leaves in areas A, B, F, and G showed a trend of first decreasing and then increasing, and the *Chlb* content at different salt concentrations was significantly lower than that in the control group (*p* < 0.05). The *Chlb* content of *S. portulacastrum* in regions K and L decreased continuously with the increase in salt concentration. In addition, the *Chlb* content of *S. portulacastrum* in region C did not change significantly before and after treatment with 200 mmol/L and 400 mmol/L salt concentrations, but decreased significantly when the salt concentration increased to 600 mmol/L. The *Chlb* content of *S. portulacastrum* in other areas increased first and then decreased.

### 3.10. Effects of Salt Stress on the Ratio of Chlorophyll a to Chlorophyll b (Rab)

The *Chla/Chlb* (*Rab*) values of *S. portulacastrum* leaves treated with different NaCl concentrations were calculated. The results showed that the *Rab* values of *S. portulacastrum* leaves of different ecotypes did not show a single change rule under different salt stress levels (Figure 8C). When NaCl concentration was 200 mmol/L, the *Rab* values of *S. portulacastrum* leaves in regions H, I, J, and K were significantly lower than those in the control group (*p* < 0.05), and the Rab values in region L were significantly higher than those in the control group (*p* < 0.05). There was no significant difference in the Rab values in other regions compared with the control group. When NaCl concentration was 400 mmol/L, the Rab value of *S. portulacastrum* leaves in area A was significantly lower than that in the control group (*p* < 0.05), and Rab values in areas B, E, G, K, and L were significantly higher than that in the control group (*p* < 0.05). There was no significant difference in Rab value between the other areas and the control group. When the salt concentration increased to 600 mmol/L, the Rab value of *S. portulacastrum* leaves in most areas was significantly lower than that of the control group (*p* < 0.05), while the Rab value in area L was not significantly different from that of the control group. Combined with the above analysis, it can be preliminarily speculated that the *S. portulacastrum* in region L has strong salt tolerance, and the *S. portulacastrum* in regions B, C, and F also have certain salt tolerance.

### 3.11. Effects of Salt Stress on Total Chlorophyll Content

The effects of different salt concentration treatments on the total chlorophyll content of *S. portulacastrum* leaves of different ecotypes from 12 regions are shown in Figure 8D. The results showed that when the concentration of NaCl was 200 mmol/L, the chlorophyll content of *S. portulacastrum* leaves in areas D, H, I, and J was significantly higher than that in the control group (*p* < 0.05), and there was no significant difference between areas E and L and the control group, while the other areas had values that were significantly lower than that in the control group (*p* < 0.05). When NaCl concentration was 400 mmol/L, the chlorophyll content of *S. portulacastrum* leaves in areas H and J was significantly higher than that in the control group (*p* < 0.05), and the other areas had values that were significantly lower than that in the control group (*p* < 0.05). When the salt concentration increased to 600 mmol/L, the chlorophyll content of *S. portulacastrum* leaves in most areas was significantly lower than that of the control group (*p* < 0.05). It can be seen that with the increase in salt concentration, the chlorophyll content in *S. portulacastrum* leaves in 12 regions showed various trends.

### 3.12. Comprehensive Evaluation of Salt Resistance Based on Principal Component Analysis

The research conducted at our university demonstrated that the combined contribution of the first two principal components accounted for 71.769% of the total variance (Table S2). Therefore, extracting these two principal components allows for a reduction in the loss of information from the original variables, leading to more satisfactory analysis results. Instead of utilizing the initial six physiological indicators (relative water content, chlorophyll content, malondialdehyde content, proline content, soluble sugar content, and plant height), we employed these two common factors to comprehensively assess the salt tolerance of *S. portulacastrum*. By employing principal component analysis, we transformed the six physiological indicators into two independent composite indicators, which can be employed for future comprehensive evaluations of *S. portulacastrum*’s salt tolerance.

We calculated and ranked the combined score (Dn values) of salt tolerance principal components for the 12 ecotypes of *S. portulacastrum*. This calculation was based on the contribution of the principal components and the physiological experimental data of these 12 ecotypes (Table S2). The results indicate that ecotypes L and I exhibit better salt tolerance, while ecotypes K, G, and J display poor salt tolerance, with the remaining ecotypes showing intermediate salt tolerance. These findings are generally consistent with the salinity tolerance ranking of 16 materials presented in previous work at our university. However, individual differences may be attributed to the inclusion of a larger sample size in Table 3.

### 3.13. Analysis of Differential Expression of S. portulacastrum Metabolism-Related Genes under Different Salt Concentrations

Generally, as long as the brightness of 28S rRNA is greater than that of 18S rRNA and the band is clear and the dispersion is slight, the integrity is good and can meet the requirements. As shown in Figure S2 from left to right three salt concentrations are shown: gully slope of Lingshui County (B) (salt-tolerant materials), Haidong Park, Binhai North Road, Dongfang City (J) (poorly salt-resistant materials), Xinglong Village, Danzhou City (L) (highly salt-tolerant materials). Electrophoresis analysis was performed on the extracted RNA samples from three areas and the three different bands of 28S, 18S, and 5S were checked (Figure S2). The RNA integrity of the 28S/18S bands was found to be between 1.9 and 2.1, indicating that the overall quality of the total RNA meets the requirements of the experiment.

### 3.14. Changes in the Relative mRNA Expression Levels of SpP5CS1, SpCHL1a, and SpCHL1b

**Figure 9:**
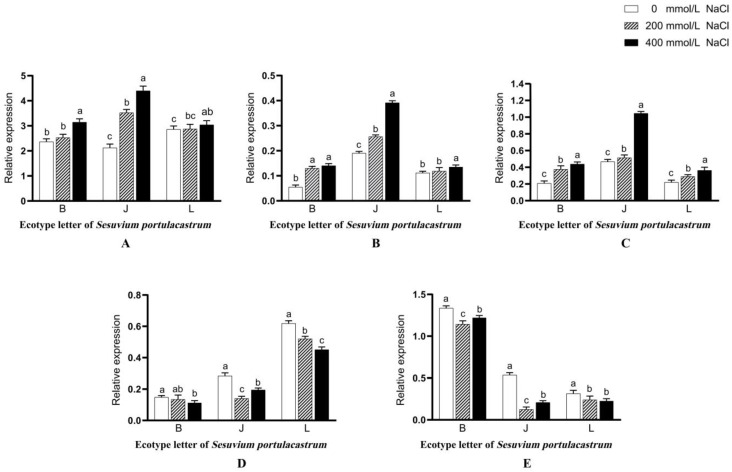
The effect of different concentrations of NaCl (0 mmol/L (blank control), 200 mmol/L, 400 mmol/L, and 600 mmol/L) on the relative mRNA expression level of *SpP5CS1* (**A**), *SpLOX1* (**B**), *SpLOX5* (**C**), *SpCHL1a* (**D**), and *SpCHL1b* (**E**). Different lowercase letters in the same group indicate significant differences at *p* < 0.05.

Compared with CK, the relative mRNA expression of *SpP5CS1* in *S. portulacastrum* in region B increased after Hoagland nutrient solution treatment with 200 mmol/L NaCl, but the change was not obvious. After treatment with Hoagland nutrient solution with 400 mmol/L NaCl, the relative mRNA expression of *SpP5CS1* began to increase significantly (*p* < 0.01). The relative mRNA expression of *SpP5CS1* in *S. portulacastrum* in region J significantly increased with the increase in salt concentration under Hoagland nutrient solution with three salt concentration gradients (*p* < 0.01). The relative mRNA expression of *SpP5CS1* in *S. portulacastrum* in region L showed an increasing trend before and after NaCl treatment with three concentration gradients, but the change was not obvious, while when comparing control and after 400 mmol/L NaCl treatment, the relative mRNA expression of *SpP5CS1* was significantly increased (*p* < 0.05) (Figure 9A).

*S. portulacastrum* in region B were treated with Hoagland nutrient solution with 200 mmol/L NaCl, and the relative mRNA expression of *SpLOX1* was significantly increased compared with CK (*p* < 0.01). When the salt concentration reached 400 mmol/L, the relative mRNA expression of *SpLOX1* had little change compared with the 200 mmol/L treatment group. The relative mRNA expression of *SpLOX1* in *S. portulacastrum* in region J was significantly increased with the increase in salt concentration in Hoagland nutrient solution treated with three salt concentration gradients (*p* < 0.01). Compared with CK, the relative mRNA expression of *SpLOX1* in *S. portulacastrum* in region L was not significantly different after 200 mmol/L NaCl treatment, while the relative expression of *SpLOX1* mRNA was significantly increased after 400 mmol/L NaCl treatment (*p* < 0.05). (Figure 9B). In addition, the relative mRNA expression of *SpLOX5* in the *S. portulacastrum* of B, J, and L showed a significant increase with the continuous increase in salt concentration (*p* < 0.01) (Figure 9C).

In the *S. portulacastrum* of B, J, and L, the relative mRNA expression levels of *SpCHL1a* and *SpCHL1b* in the treatment groups were lower than those in the CK group. The relative mRNA expressions of *SpCHL1a* and *SpCHL1b* in *S. portulacastrum* of region J decreased first and then increased with the increase in salt concentration, and the differences were significant (*p* < 0.01). With the increase in salt concentration, the relative mRNA expression of *SpCHL1a* in *S. portulacastrum* of regions B and L decreased gradually. Compared with CK and 200 mmol/L, the relative mRNA expression of *SpCHL1a* in *S. portulacastrum* in region B was significantly decreased when the salt concentration was 400 mmol/L (*p* < 0.01). However, the relative mRNA expression of *SpCHL1a* in *S. portulacastrum* in region L significantly decreased with the increase in salt concentration under Hoagland nutrient solution treated with three salt concentration gradients (*p* < 0.01). In addition, compared with CK, the relative mRNA expression of *SpCHL1b* in *S. portulacastrum* in region B was significantly decreased after salt stress treatment (*p* < 0.01), while the relative mRNA expression of *SpCHL1b* in the 400 mmol/L treatment group was significantly increased compared with the 200 mmol/L treatment group (*p* < 0.05). At the same time, the relative mRNA expression of *SpCHL1b* in *S. portulacastrum* in region L was significantly decreased in the treatment group compared with CK (*p* < 0.05), and there was no significant difference in the relative mRNA expression of *SpCHL1b* before and after the treatment of 200 mmol/L and 400 mmol/L salt concentrations (Figure 9D,E).

## 4. Discussion

Soil salinity, a significant abiotic stressor, has a notable influence on plant growth and crop yields. The stress caused by salinity can greatly impact global food production [41,42]. To date, around 10% of the Earth’s overall land area (equivalent to 950 million hectares), 20% of its arable land (about 300 million hectares), and 50% of the total irrigated land (around 230 million hectares) have been impacted by soil salinization. Moreover, it is projected to affect 50% of the world’s cultivated land by 2050, with an alarming rate of expansion [43,44]. Despite making reasonable progress in defining the physiological manifestations, the precise mechanisms underlying the response and tolerance to salt stress have remained elusive [41]. In this study, a total of 12 distinct ecotypes of *S. portulacastrum* were collected, and the changes in plant physiological indexes and the corresponding gene expression levels in response to varying salt concentrations were investigated. The results showed that various salt tolerance factors showed different changes in *S. portulacastrum* plants under different salt concentrations. Similarly, the results showed that with the increase in salt concentration, the growth rate of most *S. portulacastrum* became slow, and the growth rate of plants decreased with the increase in salt concentration. In previous studies, it has been reported that various salt concentrations significantly affected the growth and physiology of plants of different species [15,45]. Furthermore, the plant height of *S. portulacastrum* increased under low salt concentration in some areas, especially in region L. In addition, the overall internode length showed a slower growth rate with the increased concentration of salt, which corresponded with the slower growth rate of *S. portulacastrum* with the increase in salt concentration. Our results are consistent with the findings of [46] who reported that salt stress (NaCl) inhibited the growth of *Juglans microcarpa* L. seedlings. However, when the salt concentration continued to increase, most ecotypes of *S. portulacastrum* showed leaf wilting due to limited salt tolerance and the leaf area decreased sharply at this time. High salinity causes decreased plant growth primarily due to ion toxicity and nutrient imbalance [47].

Under high salt concentrations, large amounts of Na^+^ and Cl^−^ are transported to the vegetative parts of plants, which antagonistically decrease growth traits, and root and shoot lengths may be due to inappropriate Na^+^/K^+^ ratios, as higher values of Na^+^/K^+^ in plant tissue impair the transport of K^+^ and Ca^2+^, disturb plant metabolism, and lead to reduced plant growth [48]. Besides damaging the signal transduction pathway, salinity lowers osmotic potential and reduces water uptake [49]. Salinity also affects leaf initiation, internodal growth, and leaf expansion, thereby reducing shoot growth in plants [48]. Furthermore, under salinity stress, the accumulation of Na^+^ disrupts the uptake of K^+^, which reduces stomatal conductance and thus creates water-deficit conditions for plants [50]. Reduced stomatal conductance under salt stress may also lead to a reduction in photosynthetic activities [13]. Salinity also results in excessive production of reactive oxygen species (ROS) [14] and a decrease in chlorophyll contents [15]. Moreover, the presence of soluble salts in the soil elevates osmotic pressure and diminishes the overall soil water potential, resulting in reduced water absorption by the roots. Consequently, various plant growth parameters are negatively impacted [51].

The regulation of plant water content is an important part of the salt tolerance of halophytes. During salt stress, salt mainly hinders plant growth and development through osmotic stress. Under osmotic stress, the rate of decrease in relative water content is closely related to plant salt tolerance. Recent reports indicate that salinity reduces the water content in plants by impeding the absorption of water from the soil, resulting in decreased plant growth [52,53]. On the contrary, our findings revealed minimal changes in the relative water content of *S. portulacastrum* across most regions. However, in select areas experiencing low salt stress, there was an observed increase in relative water content. Typically, under salt stress, the initial physiological reaction is to prevent water loss through transpiration. This is accomplished by reducing the stomatal conductance (Gs) value through the closure of stomata [54,55]. When the salt concentration continued to increase, the relative water content of *S. portulacastrum* in most regions, except for region G, decreased significantly, which fully demonstrated that *S. portulacastrum*, as for euhalophytes, was more adapted to low salt stress conditions and even preferred the low-salt environment. Fresh weight and dry weight are the most intuitive performance indexes to determine the salt tolerance level of plants. In the current research, it was observed that a lower concentration of salt can enhance the growth of *S. portulacastrum* components, resulting in slightly higher fresh weight and dry weight compared to the control group. However, as the salt concentration increased, the fresh weight and dry weight decreased. These results indicated that high salt stress inhibited the growth of *S. portulacastrum* and significantly reduced their biomass. These findings are consistent with the results from previous studies [56,57]. Plants face difficulties in absorbing water and may even experience dehydration when subjected to NaCl treatment due to the decreased water potential of the surrounding solution. To mitigate or prevent the negative effects of osmotic stress, plants typically employ osmotic adjustment as a mechanism to restore turgor pressure [58]. Numerous experiments have demonstrated a close relationship between the salt tolerance of salt-tolerant plants and organic osmotic regulation [59,60]. Proline serves as the primary osmotic protective agent in plants when they experience salt stress. It possesses several features that contribute to its effectiveness, including its ability to prevent easy penetration of the cell membrane, lack of charge under physiological pH conditions, low molecular weight, and solubility in water [61,62]. Additionally, proline is rapidly generated within the organism and can efficiently accumulate, thereby exerting a strong regulatory effect [63,64]. The significant association between proline accumulation and osmotic stress tolerance has been extensively reported previously [61]. In our study, we observed that as the salt concentration increased, there was a noticeable increase in the proline content. We found that materials with a higher accumulation rate of proline content compared to the control exhibited greater sensitivity to salt stress. Hence, the current findings suggest that the increase in proline levels could serve as a physiological indicator for assessing the impact of NaCl on *S. portulacastrum*. Similarly, the mRNA expression of *SpP5CS1* and *SpLOX1* in *S. portulacastrum* exhibited a regulated trend after NaCl treatment with three different concentrations. In previous studies, it has been reported that delta1-pyrroline-5-carboxylate (*P5C*) synthetase and lipoxygenase (*LOX*)-related genes are significantly upregulated under salinity stress [62,65,66,67,68]. Previously, researchers reported that proline utilization pathway genes are essential for the organization and regulation of the genes involved in L-proline catabolism [69]. Proline dehydrogenase (*ProDH)* and Δ1-pyrroline-5-carboxylate dehydrogenase (*P5CDH*) are the two key enzymes in the catabolism of proline [61]. These results suggest that light regulation of salt concentration induces the expression level of *SpP5CS1* and *SpLOX1* which may maintain the salt tolerance in *S. portulacastrum*.

In living organisms, the content of MDA, the end product of lipid peroxidation, can represent the damage degree of the lipid membrane, and the change in MDA content is closely related to the permeability of membrane, in which membrane permeability directly reflects the damage degree of membrane lipid, while MDA indirectly reflects the damage degree of the membrane. In this study the vast majority of MDA contents in *S. portulacastrum* leaves did not change significantly under low salt concentration and, when the salt concentration rose, the MDA content of *S. portulacastrum* leaves of different ecotypes significantly increased to different extents. Our findings align with a previous study and indicate that salinity stress has a significant impact on the formation of malondialdehyde [70,71]. Enhanced electrolyte leakage (EL) and MDA are considered signs of membrane damage induced by salt stress [72]. Similarly, Sarker and Oba reported a similar rise in MDA in *A. tricolor* under different salt concentrations [73]. This implies that MDA is highly responsive to elevated salt stress. Chlorophyll content serves as an indicator of photosynthetic capacity and plant growth status. Specifically, chlorophyll a reflects the degree of absorption of long-wave light, while chlorophyll b facilitates the absorption of short-wave light. These functions play a crucial role in the transmission of light energy. Furthermore, this study showed that the contents of chlorophyll a, chlorophyll b, and total chlorophyll and related genes in *S. portulacastrum* of a few ecotypes in a low-salt environment were increased compared with those in the control group, while the contents of chlorophyll a, chlorophyll b, and total chlorophyll in *S. portulacastrum* of most ecotypes were decreased compared with those in the control group with the continuous increase in salt concentration. Similar results have been reported in previous studies [74,75,76]. This may be because salt stress damages the chloroplast structure of *S. portulacastrum* leaves and destroys the dynamic balance between chlorophyll synthesis and degradation, thus reducing the chlorophyll content.

## 5. Conclusions

In summary, soil salinity poses a significant threat to plant growth and crop yields, impacting global food production. With a substantial portion of the Earth’s land already affected by salinization and projections indicating further expansion, understanding the mechanisms underlying salt stress response and tolerance is crucial. In this study, we investigated the physiological and gene expression changes in *S. portulacastrum* under varying salt concentrations. Our results demonstrated that salt tolerance factors and growth parameters exhibited distinct responses to salt stress. While low salt concentrations showed positive effects on plant growth, higher concentrations led to reduced growth rates and biomass. *S. portulacastrum* exhibited different degrees of salt tolerance across ecotypes. Osmotic regulation and proline accumulation were identified as key mechanisms for salt tolerance. The increase in proline levels and the upregulation of *SpP5CS1* and *SpLOX1* genes further supported their role in salt tolerance. The MDA content reflected the damage to lipid membranes caused by salt stress. Chlorophyll content decreased with increasing salt concentration, indicating chloroplast damage and disrupted photosynthetic capacity. Overall, this study enhances our understanding of the physiological and molecular responses of *S. portulacastrum* to salt stress, providing insights into salt tolerance mechanisms and potential strategies for crop improvement in salinity-affected areas.

## Figures and Tables

**Table 1:** Ecotype Number, Longitude, Latitude, Altitude, Habitat, and Place. Habitat is related to 12 *Sesuvium portulacastrum* ecotypes.

Ecotype Number	Place	Latitude (N)	Longitude (E)	Altitude (m)	Habitat
A	Xialing Village, Qionghai City	19°23′	110°40′	30	Beach
B	Shuigoupo Village Lingshui County	18°26′	110°03′	22	Beach
C	Wanglougang Village, Ledong County	18°26′	108°51′	2	Wetland side
D	Dongjiao Port Wengtian Town Wenchang City	19°53′	110°59′	2	Bare sand
E	Hongsha Waste Water Treatment Works Sanya City	18°14′	109°33′	3	Mudflat
F	East Water Port Chengmai County	19°58	110°04′	0	Sand beside the river
G	Xingying Port Yangpu Danzhou City	19°44′	109°12′	1	Rock crevice of salt fields
H	Gangbei Port Wanning City	18°53′	110°30′	3	Beach
I	Jiangnancheng District Haikou City	20°04′	110°20′	0.4	Beach sand
J	Haidongfang Park Dongfang City	19°08	108°39′	8	Pond side
K	Yangjin Village, Lingshui County	18°26′	109°59′	1	Pond side
L	Xinglong Village, Danzhou City	19°50′	109°30′	13	Beach

**Table 2:** Growth values of internode length of *S. portulacastrum* in 12 regions after 14 days of salt stress.

Ecotype	0 mmol/L	200 mmol/L	400 mmol/L	600 mmol/L
A	0.29^a^	0.20^b^	0.04^d^	0.14^c^
B	0.38^a^	0.18^b^	0.10^c^	0.00^d^
C	0.36^b^	0.40^a^	0.04^c^	0.01^d^
D	0.56^a^	0.48^b^	0.05^c^	0.04^c^
E	0.29^b^	0.20^c^	0.23^c^	0.50^a^
F	0.74^a^	0.24^b^	0.09^d^	0.14^c^
G	0.51^a^	0.02^d^	0.06^c^	0.11^b^
H	0.33^b^	0.50^a^	0.13^c^	0.08^d^
I	0.61^a^	0.18^c^	0.02^d^	0.34^b^
J	0.13^b^	0.02^c^	0.28^a^	0.05^c^
K	0.38^c^	0.87^b^	0.25^d^	1.35^a^
L	0.09^d^	0.70^a^	0.16^c^	0.33^b^

Note: The data in the table are the mean values of internode length changes (unit: cm) after two weeks of salt stress, and different lowercase letters in the same row indicate significant differences at *p* < 0.05.

**Table 3:** The evaluation of salt resistance from 12 coastal areas of Hainan Island (A–L) based on principal component analysis.

Ecotype	Place	Overall Ratings	Rank
**A**	Xialing Village, Qionghai City	−0.218	8
**B**	Shuigoupo Village Lingshui County	0.136	6
**C**	Wanglougang Village, Ledong County	−0.274	9
**D**	Dongjiao Port Wengtian Town Wenchang City	−0.120	7
**E**	Hongsha Waste Water Treatment Works Sanya City	0.259	3
**F**	East Water Port Chengmai County	0.220	4
**G**	Xingying Port Yangpu Danzhou City	−0.925	11
**H**	Gangbei Port Wanning City	0.176	5
**I**	Jiangnancheng District Haikou City	1.561	2
**J**	Haidongfang Park Dongfang City	−0.975	12
**K**	Yangjin Village, Lingshui County	−0.858	10
**L**	Xinglong Village, Danzhou City	1.573	1

## Data Availability

Data is contained within the article or supplementary material.

## Supplementary Materials

The following supporting information can be downloaded at: https://www.mdpi.com/article/10.3390/genes14071336/s1, Figure S1: Measurement of internode length; Figure S2: Electrophoresis of total RNA; Table S1: Primers used for quantitative real-time PCR analysis.; Table S2: Loads, characteristic of roots, and contribution rates of variables in principal components.
