# Supplementary material for: Effects of Salinity Stress on Growth and Physiological Parameters and Related Gene Expression in Different Ecotypes of Sesuvium portulacastrum on Hainan Island
Source: Genes (Basel). 2023 Jun 25;14(7):1336. doi: 10.3390/genes14071336 (PMC10380013; doi:10.3390/genes14071336)
Supplement: Supplementary file 1 [file genes-14-01336-s001.zip › genes-2442733-supplementary.pdf]

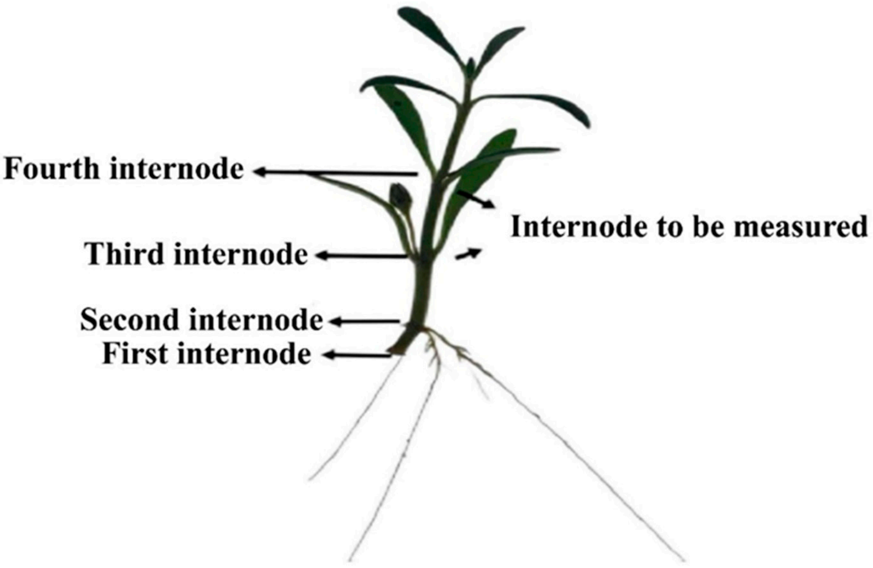

Figure S1. Measurement of internode length.

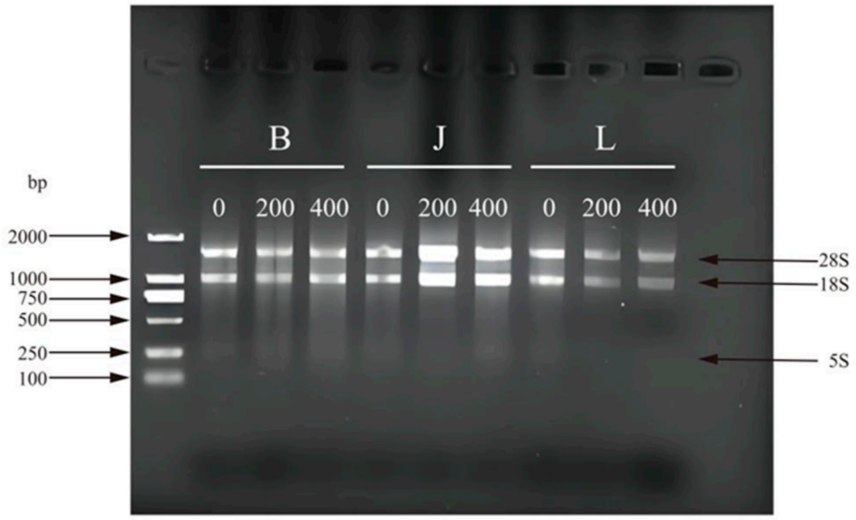

Figure S2. Electrophoresis of total RNA.

Table S1. Primers used for quantitative real-time PCR analysis.

Primer name	Primer sequence（5'-3'）	Base number (bp)
GAPDH-F	TTGGCATCGTTGAGGGTCT	19
GAPDH-R	CAGTGGGAACACGGAAAGC	19
SpP5CS1-F	TGGAACTGAAGGCCGATCTC	20
SpP5CS1-R	ATGTGTGGATGAGCTTTGATCG	22
SpLOX1-F	CAGAGTACGAGGAGCTCACGA	21
SpLOX1-R	GATCTCGTCAGAGGAATGTCTCG	23
SpLOX5-F	GTGTCTCCCTTCAGCTCATTAGT	23
SpLOX5-R	CGTCTCTCCAGCAGTCAATGA	21
SpCHL1a-F	TGGAACTGCCCACTGATTCG	20
SpCHL1a-R	TGTCCAAGAACCGGAATTGCT	21
SpCHL1b-F	ATGTTACCAATTTGGGCAACCAC	23
SpCHL1b-R	AACTTCCCATGTGACGGTTC	20

Table S2 Loads, characteristic of roots, and contribution rates of variables in principal components

Variable	Principle component 1	Principle component 2
Relative water content	-0.527	0.713
Chlorophyll content	0.785	0.277
Malondialdehyde content	0.821	0.185
Proline content	0.909	-0.302
Soluble sugar content	0.052	0.83
Plant height	0.588	0.404
Eigenvalue	2.742	1.564
Contribution rate	45.706	26.063
Cumulative contribution rate	45.706	71.769
